# “They’re always there”: resident experiences of living with rats in a disadvantaged urban neighbourhood

**DOI:** 10.1186/s12889-019-7202-6

**Published:** 2019-07-01

**Authors:** Kaylee A. Byers, Susan M. Cox, Raymond Lam, Chelsea G. Himsworth

**Affiliations:** 1 Department of Interdisciplinary Studies, University of British Columbia, Vancouver, BC Canada; 2 Canadian Wildlife Health Cooperative, Animal Health Centre, Abbotsford, BC Canada; 3 Biodiversity Research Centre, University of British Columbia, Vancouver, BC Canada; 4 School of Population and Public Health, University of British Columbia, Vancouver, BC Canada; 5 The W. Maurice Young Centre for Applied Ethics, University of British Columbia, Vancouver, BC Canada; 6 Animal Health Centre, Abbotsford, BC Canada

**Keywords:** Rats, Mental health, Public health, Social justice, Qualitative research

## Abstract

**Background:**

The presence of urban rats in the neighbourhood environment may negatively impact the physical and mental health of residents. Our study sought to describe the experiences with, perceptions of, and feelings towards rats and rat control efforts among a group of disadvantaged urban residents in Vancouver, Canada.

**Methods:**

Semi-structured interviews were held with 20 members of the Vancouver Area Network of Drug Users (VANDU) recruited by VANDU staff. Interviews were audio recorded, transcribed, and analyzed using thematic analysis.

**Results:**

Participants reported daily sightings of rats and close contact during encounters. Participants generally disliked encountering rats, raising issues of health and safety for themselves and the community due to the belief that rats carry disease. Fear of rats was common, and in some cases resulted in avoidance of rats. Effects of rats on participants were particularly pronounced for those living with rats in the home or for homeless participants who described impacts on sleep due to the sounds made by rats. Although rats were viewed as more problematic in their neighbourhood than elsewhere in Vancouver, participants believed there to be a lack of neighbourhood-level control initiatives that angered and disheartened participants. In combination with other community-level concerns (e.g., housing quality and availability), the presence of rats was viewed by some to align with a general disregard for the community and its residents.

**Conclusions:**

This study suggests that the presence of rats in urban centres may have several consequences on the physical and mental health of residents living in close contact with them. These effects may be exacerbated with continued contact with rats and when residents perceive a lack of initiative to control rats in their neighbourhood. As such, research and policies aimed at mitigating the health risks posed by rats should extend beyond disease-related risk and incorporate diverse health outcomes.

**Electronic supplementary material:**

The online version of this article (10.1186/s12889-019-7202-6) contains supplementary material, which is available to authorized users.

## Background

Neighbourhood environments are comprised of a constellation of features that influence the mental health of residents [1, 2]. For example, aspects of the built environment such as traffic level [3, 4], green space availability [5–7], and architecture [8] have been linked to psychological distress [2].

Despite the ubiquity of rats (*Rattus* spp.) in urban settings [9] their impacts on the mental health and well-being of urban residents has been understudied [10]. Rats are a potentially important neighbourhood stressor in communities where aging infrastructure, high human population density, and low socioeconomic status allow for rat populations to flourish [11–16] due to the availability of harbourage and food sources [17–19].

Rats may affect the health outcomes of residents through a number of pathways. First, rats can represent neighbourhood disorder and deterioration. Prior work has demonstrated that the perception of an area as disordered or decayed (e.g., graffiti, litter, broken glass, building abandonment, proximity to noxious land uses, and perceived violence and safety) may cause distress [20–22], and has been connected to feelings of anger, anxiety, depression, fear, powerlessness, and poorer quality of life [22–27]. Indeed, work around rat-associated psychological health impacts supports the link between mental health outcomes and rat presence [28, 29]. Because rats are more prevalent in under-resourced areas [9] where mental health effects are already particularly pronounced [8, 30], rat infestations may contribute to issues of “environmental injustice” because of greater health burdens among disadvantaged populations [31]. Second, residents may view rats negatively due to health and safety concerns [32]. Rat presence is often synonymous with disease due to rats’ carriage of numerous pathogens transmissible to people [9]. Perceived rat-associated health risks may negatively impact mental health outcomes during or following interactions with rats. For example, residents who saw rats daily were more likely to report symptoms of depression [16], while rat exposure in the home has been linked to physiological symptoms of poor mental health (e.g., headaches, dizziness, sweating, and upset stomach) [28]. This suggests that the negative effects of rat presence may be intensified for those who regularly interact with rats.

Third, because the roles of tenants and landlords in the eradication of pests are often unclear [33–35], individuals who view rat control as the landlord’s responsibility may be more likely to view rats and rat infestations as environmental hazards (i.e., threats to health and quality of life due to human action) [36]. Because harmful environmental features are recognized determinants of mental wellbeing [37] rats viewed within this context may contribute to negative mental health outcomes. Compounding this interaction, perceived inaction by landlords to address tenant complaints can result in increased stress [29]. Together, these works suggest that contact with rats in the home environment may adversely affect the mental health of residents, and that these effects can be mitigated or amplified by the actions taken by those deemed responsible (e.g., landlords, governments, or communities).

To date, there has been only one study to evaluate the impact of urban rats on psychological health [16]. Aligning with the potential pathways through which rats may affect resident health, this quantitative study demonstrated that rat exposure may act both as an independent stressor, and as a reminder of other community-level stressors, contributing to and exacerbating perceptions of neighbourhood disorder [16]. While this work has been formative in our understanding of the relationship between rats and psychological health, it does not address the breadth or extent of issues associated with rats and their impact on residents.

The aim of this qualitative study was to elicit descriptions of living with rats and to understand how these experiences affect the health and wellbeing of residents in Vancouver’s Downtown Eastside (DTES), an area where residents frequently encounter rats [38]. Regarded as Canada’s most impoverished urban neighbourhood, approximately one in every 18 people are homeless [39], and many residents live in low-income housing such as Single Room Occupancy (SRO) units [40]. In addition, there is a significant population of individuals who, due to the criminalization and law enforcement of drug use, are forced to use injection and non-injection drugs in “riskier” areas [41], potentially increasing their interactions with and exposure to rats [38]. In taking a qualitative approach, we sought to describe experiences with and perceptions of rats and rat control efforts among DTES residents. Through closer attention to the experiences of disadvantaged urban residents, it is possible to obtain a more comprehensive understanding of the effects of rats on health (both physical and psychological), which in turn will aid in devising appropriate initiatives to improve efforts to mitigate these impacts.

## Methods

This is an exploratory descriptive study [42] using individual interviews to elicit and describe the experiences of DTES residents with rats. Because of the close contact between DTES residents and rats, this area has been the site of considerable urban research evaluating urban rat ecology (e.g., [43, 44]) and health risks posed by rat-associated-zoonoses (e.g., [45–47]). The impetus for this study came from discussions with DTES community members while conducting field research on rat ecology in the area from July 2016 – January 2017. The enthusiasm with which residents shared their interactions with and feelings about rats highlighted a gap in knowledge around the potential consequences of living in close contact with rats.

To conduct this study, we collaborated with the Vancouver Area Network of Drug Users (VANDU), an internationally-recognized user-run organization that serves as a trusted community institution with over 2000 members [48]. Although VANDU’s mission is centered around improving the lives of those individuals who use drugs, members may or may not be illicit drug users [48]. We chose to recruit participants through VANDU because: 1) VANDU has extensive experience collaborating in community-engaged research [49]; and 2) because many of its members reside in Vancouver’s DTES and may have a greater exposure to rats due to issues of housing availability and quality in the DTES [40, 49] in comparison to a population with stable housing. Team members (KB and CH) have worked with VANDU on urban rat research since 2010 and have maintained ongoing communication with the Board of Directors to disseminate findings to its members. Prior to beginning the study, we met with the president of VANDU and one board-appointed member to discuss the study and seek permission to hold interviews on the premises. A pilot interview was performed with a member of VANDU to assess and inform the accessibility and appropriateness of the language and content of interview questions.

In August 2017 we held face to face interviews with 20 individuals recruited by VANDU staff. All participants in this convenience sample were members of VANDU and recruited on VANDU premises. Information about the study was provided to recruiters and made available to participants prior to the interview. Inclusion criteria was current residency in the DTES and English proficiency. Interviews were conducted in a private office at VANDU by KB and LR. Participants provided written informed consent prior to interviews and received a $10 CAD honorarium. At the start of the interview, participants were assigned a pseudonym for anonymity and were asked for their age and years of residence in the DTES. A semi-structured interview guide (Additional file 1) was used to promote discussion around participants’ experiences with rats. Some questions were predetermined (e.g.., we asked participants to describe the frequency with which they encountered rats) while others developed during interviews to explore new ideas as they arose with each participant (e.g.., in instances where participants mentioned avoiding rats, we asked participants to describe these aversion techniques). Interviews were audio recorded and lasted approximately 25 min. Field notes were kept by the interviewer (KB) to reflect on interviews following their completion.

The aims of this exploratory study were to elicit and describe the experiences of DTES residents with rats, and not to build a theory to explain these experiences. Theory-building requires a grounding of good descriptive work in order to identify and saturate the categories relevant to the theory with data [42]. Thus, we followed the descriptive qualitative content approach outlined by Sandelowski [42]. The lead researcher (KB) transcribed interviews verbatim, and transcription accuracy was assessed by LR. All transcriptions were read by KB to conceptualize emerging themes relevant to the overarching objective. Analysis was undertaken using a “thematic framework” [50]. A preliminary coding framework was developed by KB and SC for thematic analysis of transcripts. Some a priori codes were derived from the pre-determined interview questions (e.g.., timing and locations of rat encounters), and others were identified in vivo by reading through a subset of three interviews as well as field notes (e.g., activities of participants during encounters). Codes were adopted according to their relevance to the research aims and participants’ emphasis and tendency to discuss certain topics. Interviews were coded manually, and the thematic framework was revised as the analysis proceeded. Themes were developed in two ways: a priori based on the interview guide (e.g., the main theme of “Rat Encounters” was predetermined as describing these experiences was a central aim of the study); and in vivo by identifying recurring ideas relevant to the research question (e.g., one subtheme that emerged was “People Affected by Rat Encounters” as participants often mentioned that they believed certain individuals to be particularly affected by rats). Codes were grouped into main themes and subthemes that were summarized in concept maps reflecting the content of each interview; maps were then compared to derive a composite map inclusive of all themes and subthemes. Preliminary findings were presented to VANDU’s Board of Directors for member checking of the interpretation of the findings. The Board enthusiastically supported the results, and there were no modifications to the themes and subthemes presented.

## Results

### Sample characteristics

We interviewed 20 participants (five females and fifteen males). The median age of participants was 52.5 years (range: 39–69) and the median length of residency in the DTES was 12.5 years (range: 2–53). One participant identified as homeless, and one participant had been homeless previously. Ten participants explicitly mentioned living with rats, either in their home at the time of the interview (*n* = 1), in their home in the past (*n* = 7), or while homeless (*n* = 2). Throughout the results, participants are identified by gender (M for male, and F for female). Participants who identified as homeless when discussing their experiences with rats are indicated with H.

**Fig. 1:**
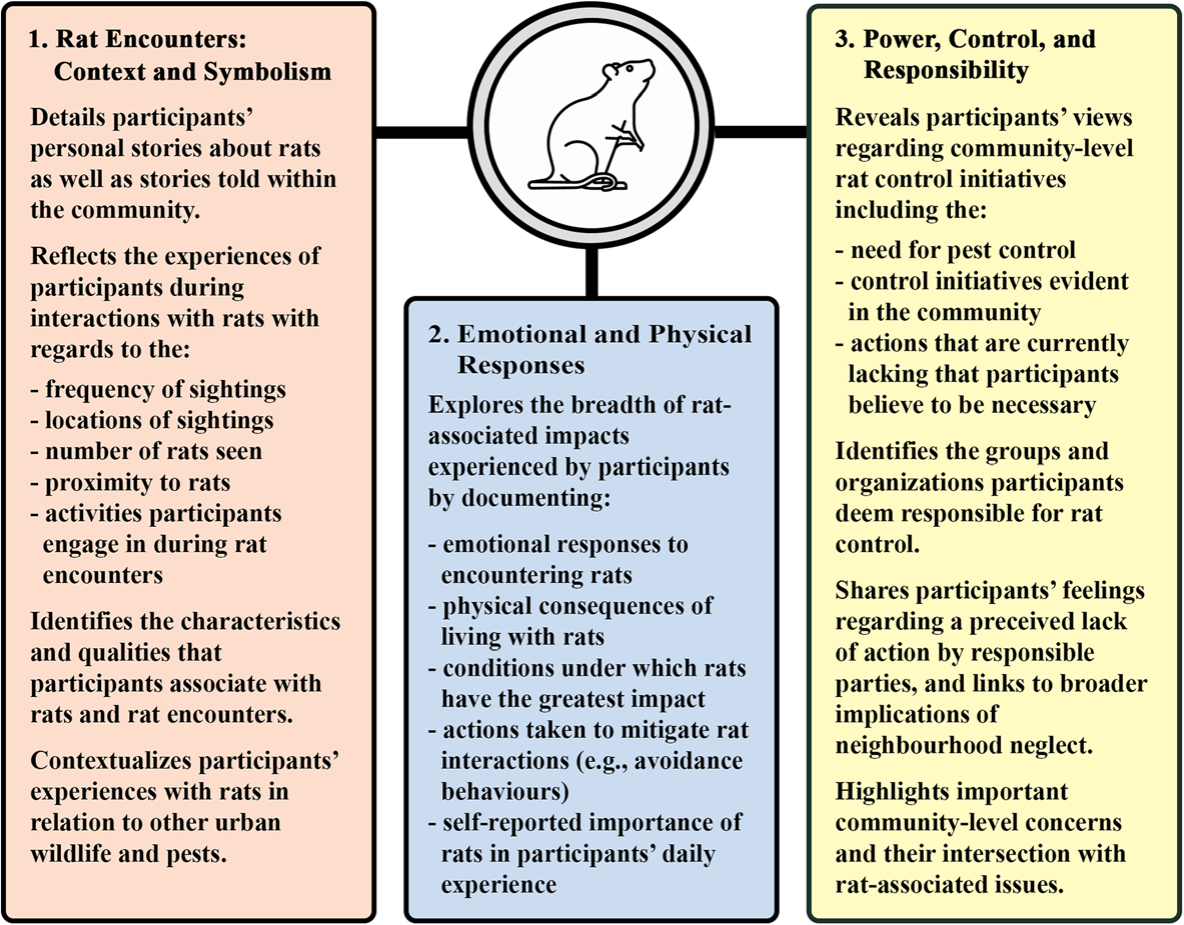
Thematic structure detailing the experiences of residents living with rats. The chief areas of description and analysis are summarized within each of the three themes: Rat Encounters: Context and Symbolism; Emotional and Physical Responses; and Power, Control, and Responsibility. Rat by Jake Dunham, available through the Noun Project

The results are presented according to three themes: Rat Encounters: Context and Symbolism; Emotional and Physical Responses; and Power, Control, and Responsibility. Figure 1 demonstrates a visual representation of the thematic structure.

### Rat encounters: context and symbolism

***“Every day I go out I see a rat, it’s a guarantee.”***

Most participants reported encountering rats daily or almost daily and described seeing anywhere from two to “hundreds” of rats regularly. Sightings were routinely in the early morning or at night, although participants were adamant that rats could be encountered at all hours. Some vigorously described the heightened sense of rat presence or abundance as troublesome.

“If you could count every goddamn rat that goes by there’re probably more fuckin’ rats around than people around the streets.” – Owen (M)

Vancouver was thought to have more rats than other Canadian cities where participants had lived (e.g., Winnipeg and Edmonton). This was attributed to an abundance of resources available to rats, specifically access to food from markets and food waste from restaurants. The warmer climate was also thought to be more hospitable to rats than other colder Canadian cities. Several participants believed that rat encounters were more frequent than approximately five to ten years prior. Participants attributed this increase to rats’ rapid reproductive rate and subsequent population growth. Only one participant believed there to be fewer rats than in the past.

“They multiply like crazy. That’s why there needs to be a handle on them. There needs to be a population control. Umm, otherwise they’ll run us over and out of town. Well, it could happen.” – Ernest (M)

Rats were considered to be “part of the DTES” due to their constant presence and prevalence. The ubiquity of rats was emphasized by almost half of participants and was particularly felt among women who stated that rats could be encountered “everywhere” in the DTES. For some, they symbolized “bad human rights”; for others, they were associated with issues of personal safety and neighbourhood disorder. Indeed, one participant indicated that the more often they saw rats, the “more dirty” they considered the city to be.

“Everything that’s in a horror movie is down here. It’s just so weird, you know? And rats – it’s interesting. They just fit right in.” – Noreen (F)

Sightings occurred in alleys, public parks, and buildings, with participants recounting stories of rats in their residence or in the residence of friends. Encounters also occurred at a local community garden and soup kitchen where participants had volunteered. However, interactions were most common in alleys, near to dumpsters, with one participant stating that rats could be seen near every dumpster in an alley. In part, it was this proximity to refuse that led participants to associate rats with garbage and being “dirty”, although this perception was also attributed to their appearance, and the smell associated with their presence.

“They’re dirty little creatures... Uhh well, they get in the garbage and everything eh? So, I don’t really care for critters like that.” – Hugh (M)

Encounters commonly occurred while walking in alleys, and some came into contact with rats while drinking alcohol or using drugs. While participants did not recount personal experiences of injecting drugs in the presence of rats, one participant felt that encountering rats was “probably a regular thing for injection users”.

“It just depends when I go hang around and go with my buddies, smoke up ‘n that, walk around with them, that’s the only time I guess I see the rats.” – Owen (M)

The most striking and recurring description of rat encounters was the proximity of rats to people and the lack of “personal boundaries”. Participants perceived rats to be “bold” and/or not “afraid of humans”, and this tendency to approach residents was viewed as bothersome and elicited fear in some participants. Other participants shared stories about encounters with rats that were vivid in detail and almost humorous in their characterizations of how the rats interacted with humans.

“I had one I was sitting on (street), there was a bag of bagels, and these bagels were like, multigrain, stale [pause] heavy, heavy things and it come out of this hole right beside me, like two inches from my body, I recognized that it was there immediately, the presence of it was so massive, and I glanced down… He crawled into this bag of bagels and with these freakin’ teeth that musta been huge, he chomped down onto this bagel, picked it up, backed out of this bag of bagels, turned around and went back in the wall. The whole time rubbing against my thigh, without an issue – without an issue. They’re just nuts.” – Ima (M, H)

The uneasiness ascribed to the proximity of rats was often linked to the belief that “bacteria” or “parasites” could be transmitted from rats to people through close contact. Despite a dearth of specific disease knowledge based in academic terms, participants were universally afraid of rat-associated diseases based on their perceived severity. Indeed, disease carriage and the risk of disease transmission to people was mentioned by nearly all participants. Although the majority were unsure of which diseases rats carried, five participants correctly cited plague and one suggested both *Escherichia coli* and *Salmonella* spp., that have been reported in rat feces [51]. Other diseases mentioned by participants, but that are not recognized-rat associated zoonoses, were rabies, malaria, and scurvy [9]. Although some participants indicated that they could not recall the disease name, they believed that the illness would present with flu-like symptoms. One participant directly linked the presence of rats to their own poor physical health.

“And they affect your breathing ‘cuz they’re shitting everywhere ‘n it gives ya flu-like symptoms right?” – Fred (M)

Rat bites were consistently cited as a route of disease transmission, either by infection with rat-associated pathogens or due to the introduction of bacteria from the environment into the wound. Participants also believed that they could contract a rat-associated illness through a scratch, contact with the rat’s fur, “droppings,” or by consuming food contaminated with rat feces. Participants were particularly concerned about food contamination in their homes, and one participant who volunteered at a soup kitchen highlighted their concern for contamination of food served to DTES residents.

“It’s just a fact of like they carry diseases and, you just never know like, one bite ‘n god knows what can happen to you… it’s just health issues... cuz they get into everything, they chew up everything and even their droppings, like you just never know like and if you miss one or something.” – Aubrey (F)

Fear of being bitten was not just associated with disease risks, but also with pain due to the strength of a rat’s bite. Some participants recounted being bitten by rats, or stories of other residents being bitten. Rat bites were often related to the tendency for rats to be aggressive, although one participant believed that these aggressive behaviours were “like any animal… even us”. In line with associations of aggression, two participants mentioned that when they thought of rats, they were reminded of the horror movies *Willard* and *Ben* from the early 1970s that involve rats attacking and killing people.

“A rat’s mouth – those teeth? They’re chewin’ through brick. So, you can imagine when they chomp on you? Wake up nothin’, you’ll be screamin’ in pain.” – Fred (M)

While rats were considered a public health concern, they were largely thought to be about as important as other pests (e.g., cockroaches and bedbugs). In fact, bedbugs were seen by some to be more concerning as they occupy the home and bite people. For example, one participant identified bedbugs as a concern when looking for housing, while none of the interviewed participants identified the presence of rats as a condition when renting an apartment. Rats may have been seen as comparable to other pests because most participants could not recount an instance where someone they knew became seriously ill from coming into contact with a rat. For example, several participants indicated that rats would gain more community-level significance if there was an outbreak of a rat-associated disease.

“I think [rats are] pretty important right now, but not more important than some of the other stuff. I think it’s important in terms of public health.” – Bruce (M)

Interestingly, several participants mentioned that rats were vilified or judged in a way that reflected how participants themselves felt judged by the Vancouver community.

“I feel like we’re being looked at like we’re overpopulated like the rats.” “It makes me feel sad. Because, the way people look down on us, they discriminate us, they judge us. It’s like, me talkin’ about rats right now, I feel like I’m [pause] judging them.” – Renee (F)

Because many DTES residents have rats as pets (either domesticated or wild caught) participants also associated rats with pets. Although pet rats were described as generally different from alley rats in appearance, only one participant expressed that it was “nice to see” pet rats. While one participant found pet rats to be “just another domesticated animal”, the majority of participants described uneasiness around them due to the potential that they “carry disease” and/or because of the supposed tendency of rats to defecate or urinate on their owners. In this way, it did not seem to matter to participants “what kind of rat it is”, with pet rats and wild rats embodying similar threats.

“Rats as, for a pet? I’m sorry. To me that is stupid. I [pause] to have a rat?... The people have them in their shirts ‘n their jackets. The rats pee and poo on ‘em. Who wants to have a pet that pees on ‘em? Poos on em? I don’t. But these people here do. I don’t get it. I don’t get it and I don’t want to get it.” – Ernest (M)

Rats were viewed as “almost the same” as other urban exploiter species (e.g., pigeons), pests (e.g., mice and cockroaches) and people. For some, the filth associated with rats and other urban animals was considered to be a result of living in the DTES and not necessarily an independent characteristic of the animal. Although parallels were drawn between mice and rats in their ability to infest buildings and carry disease, the mice’s appearance and behaviors were considered “cute” and “playful”. Participants who highlighted the commonality of rats with other animals also tended to view rats as natural parts of the environment or as serving a purpose.

“Well rats are pretty dirty, eh? Uh, but I guess everything that lives down here is, ya’know? Even the birds ‘n, and the skunks and everything that’s runnin’ around… Doesn’t really bother me… I’m sure there’s lots of ‘em that – that live here, ya’know? Even after we’re gone, they’ll still be here… there’s not too much we can do about it, ya’know? You can’t really kill ‘em all, I’m sure they serve a purpose, ya’know.” – Phil (M)

In contrast, because rats are “not native” to North America, some felt that they either “don’t belong” in the DTES, or that they were responsible for the eradication of other native species.

“Instead of chipmunks, there’s rats. Or, instead of squirrels, there’s rats.” – Noreen (F)

Participants who held this viewpoint believed that rats should be “absolutely eliminated”.

Finally, participants emphasized commonalities between the behaviors of rats and people. For example, participants told stories of rats stealing residents’ belongings (e.g., drugs), or acquiring addictions to drugs. These stories were recounted with humour and appeared to be commonly shared in the community.

“Actually, there was a picture of one… sucking on the end of the syringe... we made jokes about it all the time. Heroin rats ‘n stuff.” – Claudia (F)

“Have you heard other stories about heroin rats?” – Interviewer

“Yea. Like... they’ll come out and take peoples stuff [heroin]...” – Claudia

“Why do you think they take it?” – Interviewer

“Because they’ve already tried before, right? So [pause] they know what it is and they’re taking it just like people would do, right?” – Claudia

### Emotional and physical responses

***“Maybe people with homes, they don’t have this kind of problem.”***

Rats were considered more important for those living with them, either in their residence or when they were homeless, than for participants without rats in their home. This may be because for those without rats in their home, the distance from rats physically put them “out of sight out of mind”, whereas participants living with rats were continuously reminded of their presence. For example, one participant joked that when living with rats it was as though they were “in [the rat’s] space now, more than he’s in mine”. The invasive and pervasive nature of rats led one homeless participant to indicate that rats were the second most important factor in their life after possession theft.

“It’s exhausting. You – you never, you can never – they’re just – they’re so fuckin’ persistent they won’t leave you alone. You live on the Eastside and you live outside, bein’ homeless. I dunno, maybe people with homes, they don’t have this kind of problem. But I’m outside all the time and they’re always there. They’re always there.” – Ima (M, H)

On a physical level, participants living with rats described damage to their belongings, an inability to store food, and effects on their sleep. Participants revealed that the impact of rats at night “turns into somethin’ different”. Specifically, some participants altered where and when they slept to correspond with times that were relatively “rat free”. These changes ultimately affected the amount and quality of sleep they achieved.

“I remember gettin’ woken up and they were like, I don’t know what they were doing, they were nibbling on my hand or, ‘cuz I was sleepin’ and somethin’ woke me up, ya’know? Whether it ran by my hand or I don’t know, but, yea, they used to really bother me in there.” – Phil (M)

Impacts on sleep were attributed to the “skittering” sounds made by rats which were considered unsettling. Participants particularly affected by these sounds emphasized their volume, with one participant comparing the noises to “a six-foot male… moving furniture”. The rich description of the sounds made by rats indicated a keen awareness of their presence.

“The visual – somehow that’s expected, I don’t know. But the noises, you don’t know what it’s going to sound like, or, or the power that’s there in their little feet or something.” – Charlie (M)

Sounds made by rats were also disturbing during the day. One participant vividly recounted overhearing a mischief of rats:

“I was over by (building name) and I could hear them. Like, in the [pause] like, tons of them running across umm, the garage doors on top of the garage? Oh my god, it freaked me out, like it just freaked me out, it just ugh [pause] scared me. I thought they were gonna come falling out of the ceiling or something [pause] it was horrible.” – Claudia (F)

The participant later indicated that following this encounter she couldn’t sleep at night.

On a psychological level, the majority of participants were bothered by the presence of rats. Participants described encounters with rats as alarming, unsettling, angering, exhausting, worrisome or eliciting fear. These feelings were particularly pronounced for participants where rat encounters were “reoccurring”, and the continued alarm from confronting rats raised concerns of being bitten. In some instances, this constant exposure to rats resulted in increased sensitization to their presence such that any contact with rats elicited an immediate negative response. Indeed, the perceived consequences of living with rats went beyond direct interactions (i.e., rat bites) as described by one participant who articulated a potential cascade of negative effects from a rat infestation.

“Just how to get, how to get rid of them. What I could do to get them out of the house. Yea um [pause] and worry about is, are the walls going to start to smell, is it, you know, is this place gonna be all disgusting? If my cat gets a hold of one and eats one, will I have a sick cat on my hand? Will I get sick from touching my cat if she eats a rat? Those kind of thoughts.” – Charlie (M)

Fear was the principal feeling associated with rats and was evidenced in the characteristics attributed to them (e.g., disease and biting) and in the stories told by participants.

“I think about horror movie type things like… you’ve injured yourself and can’t move, and then rats will start eating you... I’ve heard of things like that happening down here.” – Noreen (F)

Fear was particularly acute among women. All female participants described fear of rats, and men indicated that their female friends or partners were also afraid. One male participant specifically mentioned their female friends becoming “unsure, nervous, and uncomfortable” in alleys where they had encountered rats before. One female participant who was particularly afraid of rats emphasized her inability to be alone and required that her partner carry her to the tent where they were living for fear of being touched.

“Every night I had – my boyfriend would have to carry me up the hill, because of the rats, right? I – ‘cuz I didn’t want them to touch me or anything right? I was living in a tent… I was… never alone, right? I wouldn’t sit by the door. Yea, I don’t know. I don’t know why I’m so scared of them.” – Joanne (F, H)

Anger was the second most cited emotional response to the presence of rats due to the observed impact of rats on other community members and because of the perceived inaction of responsible bodies to control rats.

“It makes me mad only that I know the reason that the rat’s usually around is that somebody’s not doing the right thing” – Mike (M)

In response to these negative feelings, purposefully avoiding rats was common among participants, but considered difficult due to their ubiquity. For some, it seemed that the only way to avoid rats was to “get out of [the] neighbourhood”. When outdoors, participants described “keep[ing their] distance” from rats by avoiding heavily infested areas or choosing to sit in areas in alleys where they were less likely to be approached by rats. Some participants described more purposeful avoidance of rats. For example, one participant mentioned that rats prevented them from participating in social activities that they enjoyed, including volunteering at a community garden and walking in alleys in the evening.

“Even when we were umm, doin’ the garden, there was rats. And I used to love working in the garden like, volunteering, but now I won’t go because of the rats.” – Renee (F)

Another participant vividly described the precautions they took to prevent contact with rats while sleeping. These precautions included hanging food, a “proximity alarm” to alert them of the presence of rats, and efforts to “cocoon” themselves inside of their blankets while sleeping, with only “a little vent hole for their mouths.”

“I had a bag of chips beside me, but I put it there intentionally because when they touch that… it makes a noise and it wakes me up and it’s like a proximity alarm right? I know they’re gettin’ close so I can, I get up and I’ll scare ‘em away... And I heard that noise. And I glanced over... And this huge cat-like creature jumped up because I scared the shit out of it, and it jumped straight up and landed on my face, then he jumped up again and he landed right back on my face then he ran down my chest, underneath the blankets, all along my thigh, and out the – out the foot of the blankets.” – Ima (M, H)

Whether bothered or not by the presence of rats, most participants believed that they had become “just the norm”.

“Well, you see them all the time, right? So basically they’re a main part of your life, right? Like, they’re everywhere. So you see them at least every hour, if not less than that, unless you leave this area. Which I don’t do a lot of... Yea they’re in my life all day long so [pause] and it’s not like you get used to it, ‘cuz you can’t get used to that, you kinda do but you don’t right? Like, it’s – nothing surprises you right?” – Claudia (F)

Some viewed this acclimatization to rats as necessary to cope with them because thinking of rats would heighten their anxiety, while others believed this approach to dealing with rats to be unnatural.

“People are so used to it already ‘cuz it’s gone so long, they don’t even – you don’t even hear anybody yell anymore. Before you used to hear a squeal, fuck! Seven years ago you used to hear girls every once in a while in the alley “Eeee!” You don’t even hear that anymore ‘cuz they’re so used to ‘em running around which is – that’s pathetic!” – Fred (M)

### Power, control, and responsibility

***“It’s not the rats that’re wrong, it’s the landlord that’s wrong.”***

Rat control was mostly thought to be the responsibility of the city, building managers, and residents, although one participant mentioned the importance of involving port officials due to the potential for rats to enter the DTES via ships.

“The city. Umm [pause] homeowners too if they have them in their back lane and they put their garbage out and they know they have rats. Anybody that’s got a rat problem should be responsible. Not just the city.” – Ernest (M)

The desire for city-initiated trapping and poisoning programs to eradicate rats was mentioned by most participants, although three participants preferred less “cruel” methods of control. Other necessary control actions were thought to be the establishment and training of a specialized “crew” of people to deal with rat infestations. Although one participant indicated that VANDU serves as a source of information on rats for community members (through their collaboration with the Vancouver Rat Project), participants underscored the importance of educating residents about rats, the diseases they carry, and how to control them.

“Like a pamphlet. I don’t see any kind of thing that say uhh ‘we have that problem down here and people need to be aware of it and this is how you deal with it.’” – Ian (M)

Improved cleanliness and the need for residents to tidy their own environments was stressed to reduce resource availability to rats. However, many did not believe that this approach would be adopted by everyone, particularly by those whose activities involved sorting through refuse for bottles or valuables. Additional methods of control mentioned were birth control and the introduction of predators.

Well I mean if people would clean up, like, clean up after themselves... I don’t think you would see that many eh? Yea. Like in the last few years, I started seeing a bit more of them because people leave their garbage behind, right? – Hugh (M)

Few participants recognized visible control initiatives in the DTES, citing poison bait boxes and metal cage traps in the alleys. It is worth mentioning that these metal traps were not part of a control effort, but instead part of the Vancouver Rat Project’s research. Indeed, four participants mentioned that they thought these traps were part of a study. Participants noted some ongoing efforts to clean the alleys and remove garbage that were thought to minimize the presence of rats. Regardless, participants felt that control efforts were insufficient.

“For one thing, they could clean up the alleys ‘n stuff more, but I know they try. See I’m making it sound like they’re not, and I know better. They try – I see it ‘n you hear the trucks all the time, but they just – they don’t have the manpower to keep up.” – Fred (M)

The majority of participants felt that there was little or no evidence of control efforts being enacted in the city. This perceived inaction was attributed to both the seemingly large population of rats and the absence of any observed control methods. Because of this, some participants revealed that they were unaware of who they could report rat infestations to.

“Who would you go to? Why wouldn’t you just deal with it yourself? [pause] I dunno, there’s not really anywhere to go is there? I dunno. I don’t think so... I assumed it was our own problem to deal with because nobody dealt with it before.” – Claudia (F)

Moreover, some participants emphasized that even if reports of rats were made to building managers, that these reports were unlikely to receive any immediate action, underscoring the perceived futility of reporting rat infestations to building managers or landlords. This contributed to feelings that dealing with rats was the tenants’ own responsibility.

“I don’t know how quick they would act on it... like in these SROs? They don’t do nothin’. ‘Cuz I’ve lived in ‘em, I lived in (location) there in – they used to run from room to room at night, ya’know? Yea, big holes, ya’know? Just goin’ room to room.” – Phil (M)

In three instances, impacts from rat infestations culminated in participants moving out of their residence. However, many implied that avoiding rats by moving was impossible due to limited availability of housing.

“Well downtown here you don’t really ask you just gotta settle with what you can get as long as you got a bed, a roof over your head it’s [pause] you just gotta take it.” – Aubrey (F)

This connection between the availability of housing and the presence of rats revealed feelings of powerlessness, hopelessness, and anger among participants who felt “stuck” in the DTES.

“Your hands are tied ‘cuz where’re you gonna live for one thing? [pause] You’re stuck here anyways in this shit, right?” – Fred (M)

Overwhelmingly, participants indicated a lack of affordable housing as the chief issue facing the community. Indeed, a number of participants emphasized their disgust with city officials for what they perceived to be “zero” action around addressing homelessness in the DTES. Further, the housing available to residents was viewed as being of poor quality. In contrast to the importance of housing, prioritizing rat control was viewed as “silly”.

“There’s so much else to deal with, like c’mon. Let’s deal with homelessness before we deal with the rat situation, seriously. Honestly, like putting money into something like that is just silly over homelessness, right?” – Claudia (F)

The intersection between a lack of action in controlling rats, and the paucity of housing were attributed to a general disregard for the DTES and its residents by the city. Participants articulated feelings that their community was being neglected, and that DTES residents “don’t count” due to homelessness or drug use. Indeed, a few participants perceived this inaction to be purposeful, and that the government was “targeting” them in order to make the land available for wealthier residents. By comparison, participants believed that similar infestations elsewhere in Vancouver would receive immediate action. When discussing this issue, many participants cited feelings of anger, while others listed hurt, sadness, depression, confusion and marginalization.

“I don’t think they think it’s that important... Well because it’s DTES right? So they really don’t care about the people down here. Because if they did they would have more housing for people, right?” – Hugh (M)

Although participants identified a need for control and were clear about who they believed to be responsible, many thought that the problem “couldn’t be fixed” due to rats’ prevalence, abundance, rapid reproductive rate, and their continuous potential to arrive to Vancouver on ships via the international shipping port. Moreover, the urgency to control rats was overshadowed by other important issues facing the DTES community such as the “fentanyl crisis,” and police harassment. When speaking of the fentanyl crisis, participants were concerned about the “safety surrounding people’s lives” and emphasized that “a lot of friends [were] gone” due to overdose deaths. One participant implied that these losses were changing the close-knit dynamics of the community. In terms of police harassment, one participant viewed police inquiry as excessive, such that residents would get “jacked up, pulled over, for standing around”, emphasizing that they felt the need to keep moving to avoid police attention. For these participants, these issues were ongoing and disruptive, whereas rats were viewed as more of a nuisance than a serious concern.

## Discussion

Our research describes the experiences of disadvantaged residents living with rats and reveals that the impacts of rats extend beyond disease-related risks. We demonstrate that chronic rat exposure may influence both the mental and physical health of residents. Notably, rats cause distress and uneasiness among those living with them, and negatively impact the sleep of residents, causing them to take precautionary measures to decrease their interactions with rats. Further, due to the symbolism surrounding rats, they contribute to views of a neighbourhood as disordered, promoting feelings of social neglect and disregard. The effects of rats arise not only due to their presence, but also from a perceived lack of action to control rats by the groups deemed responsible.

This study reveals that the public health threats posed by rats extend beyond those associated with the transmission of infectious diseases. Specifically, rats and rat-related issues can contribute to negative impacts on the mental health of residents. We document that exposure to rats was associated with feelings of anxiety, fear, and worry. In line with a quantitative study performed by German and Latkin, we found that negative perceptions of rats were not restricted to those who see rats regularly [16]. However, psychological impacts were amplified for those with daily rat exposure, especially among those who live in close contact with rats. For participants living with rats, we found that the effects were greatest at night, when rats, and the noises associated with them, disrupted participants’ sleep. The relationship between sleep disturbance and poor mental health has been discussed previously [52–54] and studies suggest that stressors have a greater impact on individuals who have poorer sleep quality [55, 56]. Thus, the effects of rats on sleep quality could exacerbate their impacts on the psychological distress of individuals already vulnerable to rats, as well as sensitize them to other environmental hazards in their community.

Rats can serve as a chronic and uncontrollable stressor for residents with frequent exposure to them. In contrast to “acute” stressors, that occur over discrete time periods (e.g., an argument with a family member), “chronic” stressors are frequent and ongoing (e.g., long-term problems with children) [57–59]. Participants in this study described everyday interactions with rats, whether while outdoors, visiting friends, or at home. Therefore, specific interactions with rats may serve as acute stressors, while continuous exposure to rats may represent a chronic stressor. This is concerning because chronic stressors may negatively affect mental health outcomes (e.g., psychological distress and depression) as much, if not more so, than acute stressors because they represent unresolved problems [58, 60]. Such unavoidable issues can promote feelings of hopelessness [61], evidenced in the ways in which participants described their inability to avoid rats without leaving their neighbourhood. Indeed, rat presence was so ubiquitous that some participants recalled seeing hundreds of rats in a single day. The pervasiveness of rats in the environment underscores issues of environmental injustice, whereby residents of under-resourced settings are disproportionately affected by stressors such as rats in comparison to more affluent communities.

To mitigate the negative effects of rats, participants employed a number of coping mechanisms. Although several participants expressed a passive acceptance of rats as part of the environment, there was no clear evidence of desensitization (i.e., that repeated rat exposure decreased their negative psychological impact). Instead, participants described a heightened awareness of rats, wherein they actively employed avoidance techniques, such as sitting in places where they were less likely to be approached by rats, spending time in alleys known to have relatively fewer rats, or employing measures to prevent contact with rats while sleeping. In some instances, avoidance was more pronounced. For example, the presence of rats prevented participants from enjoying community activities (e.g., volunteering at a local garden). While avoidance allows individuals to reduce their stress by removing the stressor from their daily experience [62], barriers to residents’ abilities to engage in social activities can potentially strengthen the effect of rats on health. This is because engaging in community-level activities can provide opportunities for social support, that in turn buffers the negative effects of stressors on mental [59, 63] and physical health outcomes [64].

Beyond the direct links between rats and the health of residents, rats represent larger community-level issues. This may be due, in part, to the stigma around rats themselves. Almost universally, participants in this study described rats as filthy and diseased, likening them to characters from horror movies. Previous work has demonstrated that the symbolism embodied by certain neighbourhood elements can signify other, more complex community-level issues. For example, Derges et al. found that the disgust associated with the presence of dog feces hints at a greater dissatisfaction with a spectrum of issues including litter, drug use, crime, and feelings of marginalization [65]. Indeed, some residents in our study likened their dislike of rats as being similar to their feelings of being stigmatized and marginalized by the greater Vancouver community. In this way, the symbolic presence of rats can contribute to and/or reflect feelings of social neglect. Similarly, rats may also denote issues of social injustice and contribute to the perception of a neighbourhood as inferior or decayed. In line with participants’ descriptions of rats as dirty, they also described their neighbourhood as filthy and neglected. In fact, many residents implied that rats were more abundant in the DTES than elsewhere in the city because of poor neighbourhood sanitation. These negative perceptions of the neighbourhood environment can also contribute to poor mental health outcomes [66]. For example, poor mental wellbeing, such as feelings of depression and hopelessness, has been linked to indices of community disorder (e.g., litter, broken glass, building abandonment etc.) [67]. As such, rats may have both a direct and indirect impact on the mental health of residents by intensifying other community-level stressors.

Mental health impacts may be compounded by the perceived inaction of responsible bodies to control them. We found that participants largely felt that rat control was the responsibility of landlords and the city, but that ultimately it fell to residents to perform their own control because they felt that neither the municipal government nor the landlords in the DTES prioritized rat infestations; this sentiment was consistent with a housing study performed in the same neighbourhood [68]. Further, participants voiced concerns that they were unable to control rats themselves, which may be due in part to the limited resources available to disadvantaged residents [69]. This view of rats as the landlord’s responsibility, in combination with feeling incapable of addressing rat-associated issues personally, suggests that rats could affect mental health as an environmental hazard [36]. Indeed, perceived inaction by those seen as responsible was met with feelings of anger, frustration, helplessness, marginalization, and sadness. These findings support previous research that found that municipal government inaction to deal with rat infestations led to feelings of helplessness among low-income residents [16], while landlord inaction increased stress by creating landlord-tenant conflicts [29]. Overall, our study revealed a strongly held belief that there was a lack of visible interest by those in positions of power to control rats due to a general disregard for the DTES and its residents. This perception appeared to contribute to adverse mental health outcomes for participants.

Rats are part of a spectrum of issues facing disadvantaged communities. Specifically, participants viewed housing availability/affordability and drug overdoses as the most significant community concerns. In contrast to the urgent nature of the housing [70] and drug overdose crises [71], rat infestations do not represent an immediate threat to residents. However, it is important to recognize that rats cannot easily be disentangled from the broader environmental health issues afflicting the community. For example, in a study interviewing female sex workers about the challenges of finding affordable housing in the DTES, individuals included rats as a contributor to deplorable and uninhabitable living conditions [69]. Downplaying the importance of rat issues in light of other societal issues is problematic because it can lead to an ineffectual response to infestations and the consequences associated with them [72].

### Strengths and limitations

This study has several limitations to be considered. First, our sampling of participants was restricted to members of a community-based organization, preventing us from making generalizations about the experiences of all residents in the DTES, or to residents of other low-income areas. Second, due to the sampling of our study, we are unable to offer rigorous comparisons about differences in experience arising in the data according to participant characteristics such as gender and housing status. For example, of the participants interviewed, five of 20 were women, while only two of 20 identified as living with rats while homeless. Given that women in our study emphasized feelings of fear and discomfort around rats, and that male participants also identified feelings of fear among their female partners and/or friends, further focus on how individuals experience rats in relation to gender is an important area for future research. Similarly, that participants identifying as homeless considered rats to be more important to them than did those with homes suggests that future research in this area would benefit from explicit and purposive sampling of homeless persons.

Despite these limitations, this exploratory study is the first to our knowledge to qualitatively describe the experiences of residents with rats and strengthens the growing body of literature suggesting that interactions with rats can negatively impact the mental health of residents [10]. Given this association, we suggest that future work build upon these findings to gather data which addresses the underlying pathways through which rats impact the health outcomes of residents, as well as how these interactions relate to other environmental hazards experienced in urban settings.

## Conclusions

This study supports the need to re-evaluate the ways in which we conceptualize the consequences of living with rats to incorporate diverse health outcomes. While the chronic nature of rat infestations in some low-income city settings may diminish the importance placed on their control, their role as an independent stressor and their contribution to other neighbourhood-level issues necessitates proactive approaches to rats. As such, integrated approaches engaging governments, landlords, and communities is necessary to monitor and mitigate the impacts of rats in the urban environment and promote effective control initiatives.

## Additional file

Additional file 1: Interview Guide. Provides a list of questions used to guide interviews with participants regarding their experiences living with rats. (DOCX 15 kb)

## Data Availability

The transcripts generated from interviews in this study are not publicly available because prior consent from participants was only obtained for the research team to have full access.

## Abbreviations

DTES: Downtown Eastside
VANDU: Vancouver area network of drug users
VRP: Vancouver rat project
