# Supplementary material for: “They’re always there”: resident experiences of living with rats in a disadvantaged urban neighbourhood
Source: BMC Public Health. 2019 Jul 1;19:853. doi: 10.1186/s12889-019-7202-6 (PMC6604318; doi:10.1186/s12889-019-7202-6)
Supplement: Supplementary file 1 — Interview Guide. Provides a list of questions used to guide interviews with participants regarding their experiences living with rats. (DOCX 15 kb) [file 12889_2019_7202_MOESM1_ESM.docx]

**Additional File 1: Interview Guide**

How long have you lived in the Downtown Eastside (DTES)?

How often do you see rats? (e.g., every day, several times a week, once a week, once a month or never?)

Can you describe a typical encounter you might have with rats?

(*Prompts: Where, when*)

Can you describe a memorable encounter with rats you had, or heard of?

*(Prompt: Are there any stories about rats that are told often in the community?)*

When you see rats like this, how does that make you feel? Why?

Do you change your habits around rats?

What impacts do rats have on the community? What do they mean for the community?

Aside from the feelings you just said, do rats have any other meanings for you?

Are there any other issues associated with rats from your point of view?

Who do you think should be responsible for dealing with rats in the DTES?

Do you think rats are a bigger problem in the DTES than other parts of Vancouver?

Have rats affected where you live or how you feel about where you live?

Of all the things that you deal with in your day, how important are rats? Why do you think that?

*(Prompt: Are they the least important? Most important? The same as other things?)*

I have asked you a lot of questions today. Is there anything else you would like to share with me about your experiences with rats in the DTES?
